# State Provision for Inebriates and Drug Habitues

**Published:** 1907-07

**Authors:** C. C. Stockard

**Affiliations:** Atlanta, Georgia


### STATE PROVISION FOR INEBRIATES AND DRUG HABITUES.

By C. C. Stockard M. D., Atlanta, Georgia.

Few people have any idea of the extent to which the use, as a habit of some drugs, more especially opium and morphine, has grown, and, of the wreck and ruin it is causing. The Alcoholic cannot so easily hide his addiction, the symptoms being more marked and generally known.

It will doubtless be a surprise to most of you to hear that the amount of opium and morphine brought into the United States annually is five or six times what it was six years ago, averaging more than sixty grains of morphine to every man, woman and child, while in China only twenty-seven grains are used, and in Europe and South America only twelve. While this enormous amount is being consumed annually, I am quite sure that the legitimate use has diminished, at least proportionately. Though this is true, it is still a fact that a large proportion of the cases of addiction results from being used first by the physician, and therefore, the double responsibility rests upon us to do what we can to check the evil, and also to rescue those who have already fallen victims.

Right here let me say a few words on the attitude of a large portion of the profession, as well as the laity, toward the users of drugs. I find that a large majority are without sympathy for them and feel that those people simply want to keep up the use of drugs for the good feeling it gives them, and really have no desire of quitting the use. This, I am confident, does these poor unfortunates a great injustice. Look around you and think of the men you know, bright, intelligent, highly educated, but insiduously been led on to the use of alcohol or morphine, either to enable them to accomplish some important task, or, to relieve pain that they might proceed to the performance of duties pressing upon them, and who have finally become so weakened by nervous debility that they have lost control of themselves and are absolutely helpless against their master. Tell me these men would not like to go back to their former conditions? They all realize their wrecked state, and make effort after effort to get relief, but in a large proportion of cases, fail, because they cannot afford the time and money for sufficiently prolonged treatment and rest, to enable them to be restored to something like their normal condition. The following letter, received by me a few months ago from a bright, intelligent, young physician, illustrates this. He is now of his own volition, in the State Sanitarium.

"Dear Dr.:—

I am writing to ask you to do me a favor, which I am sure you will take pleasure in doing for me.

I am sorry to have to admit it, but have again, in fact, I have been fooling with Morphine ever since you treated me last, but up until Christmas I did not take up the "habit" again regularly. I would take two or three doses one day and then leave it off entirely for three or four days, consequently I was nervous and depressed all the time and not fit to do any work at all. I had to take the Morphine occasionally to enable me to leave off whiskey, and I left it off entirely until Christmas eve night. I drank about a quart of whiskey in a few hours and took a number of hypodermics of Morphine afterwards. I do not know how much I took, for I was too drunk to realize what I was doing, but must have taken several grains. Any way I was crazy as a bat for several days. Have been using Morphine regularly since, am now taking about 30 grains hypodermically daily.

Dr., I realize that I never will quit whiskey and morphine without help, so I have given my consent to go where I can not get hold of either, and stay until I am safe—three, six—twelve months or longer.

A jury will try me tomorrow (Saturday), and if possible I will be sent to the asylum at Milledgeville on charge of Dipsomania. The papers will be sent to Milledgeville Sunday or Monday.

Dr., won't you write a personal letter to Dr. Powell at the asylum and explain my case thoroughly to him, and see if you can't help me to get in, for if I don't get there I am ruined for life—I haven't any money—can't borrow any—and have lost all my practice and everbody around here knows of my habit now. Thanking you in advance I remain— Your friend

Please be sure to write to Dr. Powell by return mail—if you do not I am afraid your letter will reach him too late."

Of the second class above mentioned, viz:—those who begin by taking opiates to relieve them of pain to enable them to do urgent work, whose own profession furnishes the large majority, and it is pitiful enough that one, who for the sake of doing good to others, is led to do that for himself which carries him to his utter ruin.

The practical question is, what can be done? And it is to the answer to this question that I wish to call your attention, and hope to enlist your interest. The State provides for its insane, its feebleminded, its criminals, and why not for its alcoholic and drug habitues who are often as dangerous to society as the insane, or criminal, and as dependent as the feeble-minded or the blind?

It is recognized by all who have studied the matter, that Alcoholism, Morphinism, etc., are real diseased conditions, or the results of them, though many of the uninformed still hold to the old idea and think punishment the proper remedy. This is perfectly natural, and it is always hard to controvert prevalent ideas, no matter how erroneous they may be. Great progress has been made, however, during the past 25 years, and, as a result, a number of Institutions have already been established, and other States will fall into line as the people become better informed. What I am saying applies to the Habitues of Alcohol, as well as other drugs, and what is needed in my opinion, is, that each State should establish a Sanitarium, to which patients can commit themselves, or be committed by family or friends, without having to go through the legal processes at present necessary, to enforce the sufficiently long detention or restraint to get permanent results. At present, one can only be legally restrained by going through the process of being adjudged insane, which can not be done except in extreme cases; or, of having a guardian appointed, which is just about as objectionable.

The thing needed is, to be able to commit one on proof of habitual drunkenness, or, habituation to any drug which incapacitates, or makes one dangerous to his family or the public. As I said above, the majority of these patients are unable to stand the expense of sufficiently prolonged confinement in an Institution; and besides, they will not do it, even if they could, for the reason that they always feel, after being off their accostomed drug a little while, that they are safe. They all say, "If I am once free from it, I'll never touch it again", and they firmly believe it, while we know that few are permanently relieved. On the contrary, when they are using the drug and feel their own inability to free themselves, they will do almost anything on the hope of getting free and I am sure many of them would voluntarily commit themselves to an Institution where they felt that humane treatment would be given them, as in the case above referred to.

The ideal Institution for this work, it seems to me, should be located several miles from any town or city, should be arranged to give good accommodations to those able to pay for it, as well as taking care of the indigent. There should be arrangements for healthful exercise and amusement, and also a library, as many of these patients are among the best educated and most intelligent. Commitment should be, for not less than six months, and usually a year or more.

Such an Institution in every State, and admitting persons addicted to alcoholics or drugs, upon proof that their habit incapacitates them, would in my opinion accomplish great good.

I think that it would probably be well to have minor cigarette smokers included among those eligible to commitment. Something, at any rate, should be done to diminish this evil, which is sapping the manhood of our boys, and is the foundation for development of Neurotics; Morphine, Alcohol or Cocaine habitues. A large majority of the boys who begin smoking cigarettes at ten, will at fourteen be taking beer, and spend their lives the victims of Alcohol or Opium.

The establishment of Institutions on some lines as above indicated is being considered in many of the States, and all must come to it sooner or later. The medical profession can do more toward bringing about the result than any other class, especially in developing sentiment in that direction. And I am sure that we cannot spend our energies where more needed, for certainly in our country these evils are causing untold anxiety, distress and misery.

When some measure looking to the establishment of an Instiution for the treatment of these cases is proposed in our legislature, let us help it on in every way possible, and in the meantime do what we can toward turning public sentiment in that direction.

30 Crew Street.

